# Detection of gene mutations and gene–gene fusions in circulating cell‐free DNA of glioblastoma patients: an avenue for clinically relevant diagnostic analysis

**DOI:** 10.1002/1878-0261.13157

**Published:** 2022-02-11

**Authors:** Vikrant Palande, Tali Siegal, Rajesh Detroja, Alessandro Gorohovski, Rainer Glass, Charlotte Flueh, Andrew A. Kanner, Yoseph Laviv, Sagi Har‐Nof, Adva Levy‐Barda, Marcela Viviana Karpuj, Marina Kurtz, Shira Perez, Dorith Raviv Shay, Milana Frenkel‐Morgenstern

**Affiliations:** ^1^ Azrieli Faculty of Medicine Bar‐Ilan University Safed Israel; ^2^ Neuro‐Oncology Center Rabin Medical Center Petah Tikva Israel; ^3^ Hebrew University Jerusalem Israel; ^4^ Department of Neurosurgery Ludwig‐Maximilians‐University Munich Germany; ^5^ Department of Neurosurgery University Hospital of Schleswig‐Holstein Kiel Germany; ^6^ Department of Neurosurgery Rabin Medical Center Petah Tikva Israel; ^7^ Sackler School of Medicine Tel Aviv University Tel Aviv Israel; ^8^ Biobank, Department of Pathology Rabin Medical Center Petah Tikva Israel; ^9^ The Dangoor Centre For Personalized Medicine Bar‐Ilan University Ramat Gan Israel

**Keywords:** circulating cell‐free DNA, druggable, gene mutation, gene‐gene fusion, glioblastoma, liquid biopsy

## Abstract

Glioblastoma (GBM) is the most common type of glioma and is uniformly fatal. Currently, tumour heterogeneity and mutation acquisition are major impedances for tailoring personalized therapy. We collected blood and tumour tissue samples from 25 GBM patients and 25 blood samples from healthy controls. Cell‐free DNA (cfDNA) was extracted from the plasma of GBM patients and from healthy controls. Tumour DNA was extracted from fresh tumour samples. Extracted DNA was sequenced using a whole‐genome sequencing procedure. We also collected 180 tumour DNA datasets from GBM patients publicly available at the TCGA/PANCANCER project. These data were analysed for mutations and gene–gene fusions that could be potential druggable targets. We found that plasma cfDNA concentrations in GBM patients were significantly elevated (22.6 ± 5 ng·mL^−1^), as compared to healthy controls (1.4 ± 0.4 ng·mL^−1^) of the same average age. We identified unique mutations in the cfDNA and tumour DNA of each GBM patient, including some of the most frequently mutated genes in GBM according to the COSMIC database (*TP53*, 18.75%; *EGFR*, 37.5%; *NF1*, 12.5%; *LRP1B*, 25%; *IRS4*, 25%). Using our gene–gene fusion database, ChiTaRS 5.0, we identified gene–gene fusions in cfDNA and tumour DNA, such as *KDR*–*PDGFRA* and *NCDN*–*PDGFRA*, which correspond to previously reported alterations of *PDGFRA* in GBM (44% of all samples). Interestingly, the PDGFRA protein fusions can be targeted by tyrosine kinase inhibitors such as imatinib, sunitinib, and sorafenib. Moreover, we identified *BCR*–*ABL1* (in 8% of patients), *COL1A1*–*PDGFB* (8%), *NIN*–*PDGFRB* (8%), and *FGFR1*–*BCR* (4%) in cfDNA of patients, which can be targeted by analogues of imatinib. *ROS1* fusions (*CEP85L*–*ROS1* and *GOPC*–*ROS1*), identified in 8% of patient cfDNA, might be targeted by crizotinib, entrectinib, or larotrectinib. Thus, our study suggests that integrated analysis of cfDNA plasma concentration, gene mutations, and gene–gene fusions can serve as a diagnostic modality for distinguishing GBM patients who may benefit from targeted therapy. These results open new avenues for precision medicine in GBM, using noninvasive liquid biopsy diagnostics to assess personalized patient profiles. Moreover, repeated detection of druggable targets over the course of the disease may provide real‐time information on the evolving molecular landscape of the tumour.

## Graphical abstract

Personalized therapy of patients with glioblastoma (GBM) is challenging owing to tumour heterogeneity. Here, we extracted and sequenced cell‐free DNA (cfDNA) from the plasma of 25 GBM patients and tumour DNA from fresh tumour samples. We found that cfDNA concentrations in the plasma of GBM patients were significantly elevated, as compared to healthy controls. Moreover, we identified unique mutations and gene–gene fusions in the cfDNA and tumour DNA of GBM patients, some of which could be therapeutically targeted by tyrosine kinase inhibitors.

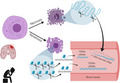

**Abbreviations**
cfDNA: cell free DNA
ChiPPI: chimeric protein‐protein interactions
ChiTaRS: chimeric RNAs and RNA‐seq database
ctDNA: circulating tumour DNA
DNA‐seq: DNA sequencing
GBM: glioblastoma multiforme
NGS: next generation sequencing
RNA‐seq: RNA sequencing

## 1 Introduction

Gliomas are primary brain tumours that account for about 30% of central nervous system tumours and for 80% of malignant brain tumours [1]. Glioblastoma (GBM) is the most common type of glial tumour and is uniformly fatal [2], with a median survival time of only 12–15 months [3, 4]. Diagnosis requires evaluation by magnetic resonance imaging (MRI), followed by tissue examination attained either by biopsy or during surgical resection of the tumour. In about 40% of GBM cases, the *O* ^6^‐methylguanine DNA methyltransferase (MGMT) promoter is methylated, rendering the tumours more susceptible to temozolomide, an alkylating agent that methylates DNA, and which constitutes standard chemotherapy [5]. Current methods for tumour monitoring (e.g., MRI and computed tomography [CT]) cannot provide real‐time actionable information for determining therapy responses or for following the evolving molecular landscape of the heterogeneous tumour cell population [6]. In contrast, a liquid biopsy platform that considers circulating cell‐free DNA (cfDNA) may overcome limitations associated with glioma heterogeneity, could provide a means for diagnosis, and possibly guide precision medicine for patients [7].

Liquid biopsy is an emerging noninvasive cancer diagnostic technique that potentially provides an alternative to repeated surgical biopsies. Liquid biopsy provides information on a tumour derived from simple blood, urine, saliva, or other body fluids samples [7, 8, 9, 10]. Cellular elements are released from the tumour and healthy tissues into the bloodstream as a result of secretion, apoptosis, and/or necrosis [10, 11] and can be screened for tumour‐specific markers that may be useful in diagnosis, monitoring, treatment decision, or prognosis [12]. However, given the unique architecture of the brain, it has been demonstrated that levels of detectable cfDNA in brain tumours are reduced by 60%, and by 90% in medulloblastoma and in low‐grade glioma, respectively, as compared to various systemic malignancies [13]. Thus, detecting cfDNA in glioma patients for clinically relevant purposes remains a challenging and complex problem.

**Fig. 1:**
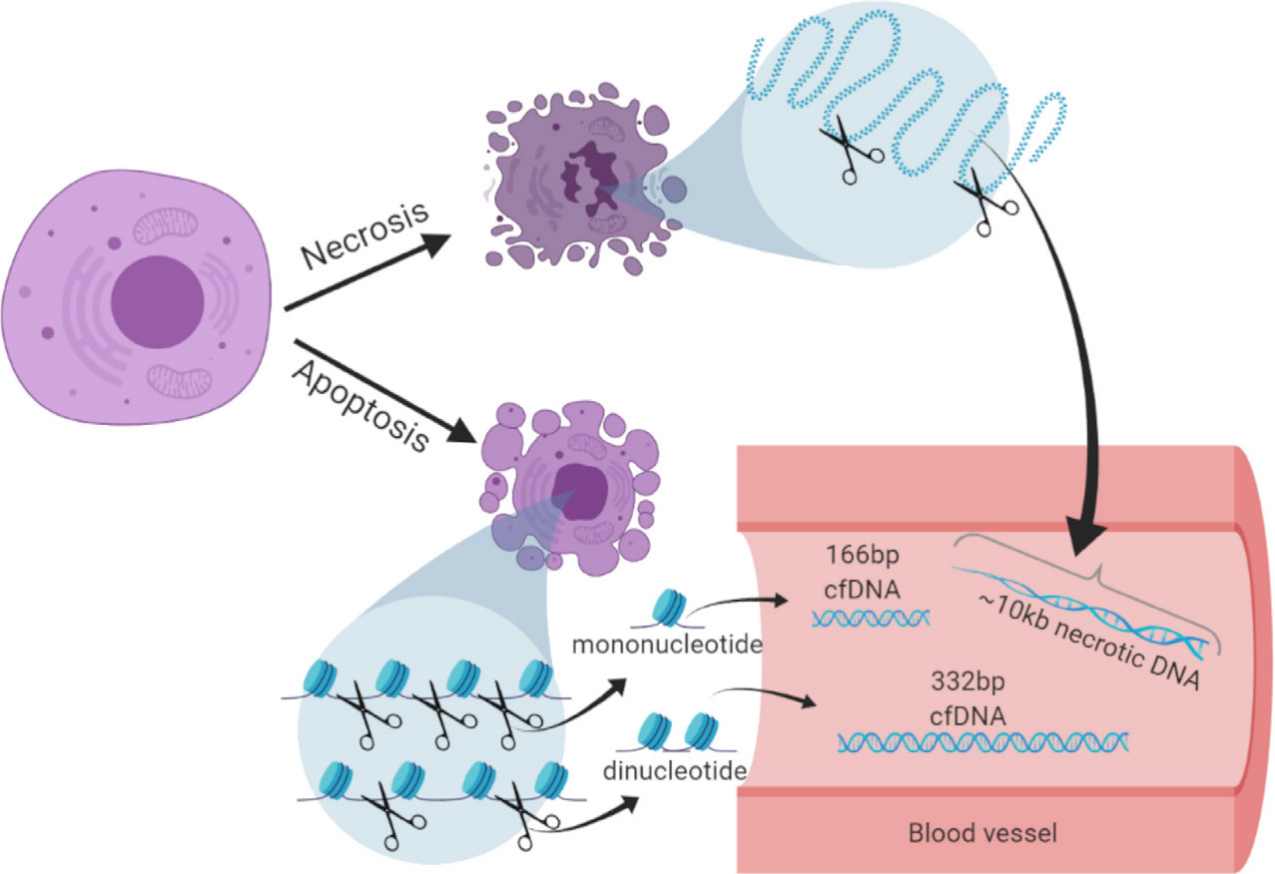
Schematic representation on the origin of different cfDNA size detectable in blood following cellular apoptosis and necrosis. Cells undergoing apoptosis and necrosis release their nuclear DNA that is fragmented in the circulation around nucleosomes in the case of the apoptosis, but random long fragments in the case of necrosis. The different fragment sizes of cfDNA circulating in blood are usually 166 bp, 332 bp, and 448 bp from the apoptosis process and > 1000 bp from the necrosis process [11, 52, 61]. These sizes have been observed in many previous studies as well as in our study.

cfDNA constitutes free‐floating small fragments of DNA in blood plasma, which result from apoptotic cell death [10, 11]. Remarkably, elevated levels of cfDNA have been documented in solid tumours, including some gliomas, relative to patients with non‐neoplastic diseases [9, 10]. Of cfDNA fragments present in cancer patient plasma, 85% are 166 bp, 10% are 332 bp, and 5% are 498 bp in length [7, 13]. In contrast, larger cfDNA fragments (~ 10 000 bp in length) detectable in cancer patients are most likely the products of necrosis [8, 9, 13] (Fig. 1).

Chromosomal aberrations play a crucial role in tumorigenesis [14, 15, 16, 17, 18, 19, 20]. This is especially true for chromosomal translocations and their corresponding gene–gene fusions, which disrupt cellular regulatory mechanisms [15, 16, 17, 18, 19]. For example, *TMPRSS2‐ERG* fusion genes have been detected in 40–80% of prostate cancers [21, 22]. The *BCR‐ABL* fusion gene is most commonly observed in chronic myelogenous leukaemia [23, 24]. Overall, around 90% of lymphomas and nearly half of all forms of leukaemia harbour translocation‐induced gene fusions [19, 21, 25]. Thus far, gene–gene fusions in malignant gliomas have not been thoroughly investigated.

In our Chimeric Transcripts and RNA‐Seq (ChiTaRS‐5.0) database, we collected more than 40 000 unique fusion transcripts from more than 40 cancers [26]. This represents the largest collection of chimeric transcripts of chromosomal translocations and RNA trans‐splicing cases in cancer currently available [19, 27, 28, 29]. Moreover, we collated data on about 200 unique druggable fusion genes from PubMed articles using our text‐mining method ProtFus [20]. In the present study, we sequenced cfDNA from 25 GBM patients and assessed plasma concentrations, mutation patterns, and novel druggable fusion genes encoding products that can potentially be targeted by crizotinib and imatinib analogues. All the results were compared with the findings of similar analysis of 180 tumour DNA samples of patients from the TCGA/PANCANCER project and by text‐mining of PubMed papers using ProtFus. Our findings may thus help guide precision medicine for GBM‐tailored therapy.

## 2 Materials and methods

### 2.1 Sample collection, storage, and maintenance

Brain tumour samples (freshly frozen), blood plasma, and peripheral blood mononuclear cells (PBMCs) were obtained from 25 glioblastoma patients treated at several hospitals and from biorepository samples. Nine samples were provided by Dr. Charlotte Flueh, Department of Neurosurgery, University Hospital of Schleswig‐Holstein, Campus Kiel, Kiel, Germany, ten samples were provided by Prof. Tali Siegal, Neuro‐Oncology Center, Rabin Medical Center, Petah Tikva, Israel, and six samples were provided by The Israeli National Tissue Bank (MIDGAM). We collected blood samples that were separated into plasma and PBMCs from 25 healthy donors of similar ages without current/previous cancer diagnosis. Blood was collected into EDTA‐coated anticoagulation tubes, and plasma was separated within 2 h of collection. About 1–2 mL of plasma and about 1 mL of PBMC were separated from each blood sample. Both samples were kept at −80°C and shipped on dry ice. The research was approved by the Ethics Committees of the Rabin Medical Center, Israel, on February 12, 2017 (ethic code: 0039‐17‐RMC) and by the Faculty of Medicine, Der Christian‐Albrechts‐Universität zu Kiel, Germany, on February 26, 2015 (ethic code: D 405/14). The experiments were undertaken with the understanding and written consent of each subject. The study methodologies conformed to the standards set by the Declaration of Helsinki.

### 2.2 DNA isolation

cfDNA was isolated using a QIAamp Circulating Nucleic Acid Kit (Qiagen, Chatsworth, CA) from different volumes of plasma samples (850 µL to 2 mL). All samples were processed according to the manufacturer's standard protocol. A NucleoSpin Tissue Kit (Macherey‐Nagel, Duren, Germany) was used to process genomic DNA from 25 mg of brain tumour biopsies and from 0.5 mL of PBMCs from each patient. Isolated DNA samples were stored at −20°C until further use.

### 2.3 DNA quantification

All isolated DNA samples were quantified by a Qubit dsDNA High Sensitivity assay (ThermoFisher Scientific, Waltham, MA) using a Qubit2.0 fluorometer. The assay was performed according to the manufacturer's standard protocol. Fluorescence was measured at 485/530 nm to determine DNA concentration for each sample. A Bioanalyzer 2100 DNA High Sensitivity assay was performed to determine fragment size distribution in isolated cfDNA samples.

### 2.4 Next‐generation sequencing and data analysis

A NEBNext Ultra II DNA Library Prep Kit (New England Biolabs, Ipswich, MA, USA) was used for NGS library preparation. Sample libraries were sequenced on Illumina HiSeq 2500 and Illumina NextSeq 550 (San Diego, CA) platforms at the Genomic Center of the Azrieli Faculty of Medicine, Bar‐Ilan University. To avoid batch effects, samples were assigned different lanes, and positioned within cfDNA, tDNA, and gDNA samples of different patients. The COVARIS fragmentation step was performed only for tDNA and germline DNA from PBMCs. All samples from GBM patients were sequenced by paired‐end 100 bp whole genome sequencing at an average of 30X coverage (cfDNA, gDNA, and tDNA). NGS data were subject to quality control analysis of raw sequencing reads using FastQC and an additional in‐house shell script. Adapters and low‐quality sequences were trimmed using Cutadapt (113). Remaining reads were mapped to a human genome reference (hg38) using Bowtie2 [30, 31, 32] and SAMtools [30].

### 2.5 SNV (single nucleotide variant) analysis

SNVs in each sample were identified using BCFtools mpileup [30]. Bioinformatics analysis of SNVs was performed.

### 2.6 Fusion genes analysis

For fusion gene analysis, reads that did not map to the reference human genome (hg38) were extracted using SAMtools [30]. These were instead mapped against the reference database of unique chimera junction sequences ChiTaRS‐5.0, using an in‐house chimera search algorithm [33].

### 2.7 Gene set enrichment analysis

Gene set enrichment analysis was performed using ‘webgestalt’[34, 35], in which two gene sets (i.e., a set of genes commonly mutated in GBM and a set of genes that fused with high frequencies in GBM tumours and cfDNA) were analysed against the KEGG pathway database [36, 37, 38]. The 100 most significant pathways connected for the genes in each gene set were compared to identify pathways common to the sets.

### 2.8 Mutation validation using Sanger sequencing

Twenty‐two‐point mutations from tumours and cfDNA from GBM patients were selected for validation by Sanger sequencing. Primers were designed using Primer3 (v. 0.4.0) [39, 40]. All amplified polymerase chain reaction (PCR) products were isolated using silica membrane spin columns (NucleoSpin Gel, PCR clean‐up kit) and were eluted in 20 µL of nuclease‐free water. PCR products were then processed for Sanger sequencing and the results were analysed using the Basic Local Alignment Search Tool (BLAST) and Chromas 2.6.2 (Technelysium, South Brisbane QLD, Australia, accessed on Dec 4, 2019).

### 2.9 SNV analysis

SNVs were identified using bcftools mpileup for each sample (106). "GB13" patient was used for an example below.

Step 1: Mapping of trimmed to hg38 reference genome:

Mapping of trimmed reads to human reference genome (*hg38*) was performed using *bowtie2* with default parameters that sends results into a BAM file for each sample.

Step 2: Generates genotype likelihoods at each genomic position with coverage

*‘bcftools mpileup’* was used to generate genotype likelihoods at each genomic position with coverage from the BAM file for each sample without indel.

e.g., *bcftools mpileup ‐I ‐Ou ‐f hg38.fa GB13_Tumor.bam*

Step 3: Actual calling of SNVs:

*‘bcftools call’* was used with option ‐m (alternative model for multiallelic and rare‐variant calling) to call SNVs for each sample.

e.g., *bcftools mpileup ‐I ‐Ou ‐f hg38.fa GB13_Tumor.bam* | *bcftools call ‐mv ‐Ou*

Step 4: Normalization of a variant:

Normalization of called variants was performed using *‘bcftools norm’* for each sample.

e.g., *bcftools mpileup ‐I ‐Ou ‐f hg38.fa GB13_Tumor.bam | bcftools call ‐mv ‐Ou | bcftools norm ‐Ou ‐f hg38.fa*

Step 5: Filtering the SNVs

Finally, raw SNPs were filtered using *‘vcfutils.pl varFilter’* for each sample with default parameters to generate a final filtered VCF file.

e.g., *bcftools mpileup ‐I ‐Ou ‐f hg38.fa GB13_Tumor.bam | bcftools call ‐mv ‐Ou | bcftools norm ‐Ou ‐f hg38.fa* | *bcftools view* | *vcfutils.pl varFilter ‐ > GB13_Tumor.vcf*

*‘vcfutils.pl varFilter’ by default using following parameters:*

*‐Q INT minimum RMS mapping quality for SNPs [10]*

*‐d INT minimum read depth [2]*

*‐D INT maximum read depth [10000000]*

*‐a INT minimum number of alternate bases [2]*

*‐w INT SNP within INT bp around a gap to be filtered [3]*

*‐W INT window size for filtering adjacent gaps [10]*

*‐1 FLOAT min P‐value for strand bias (given PV4) [0.0001]*

*‐2 FLOAT min P‐value for baseQ bias [1e‐100]*

*‐3 FLOAT min P‐value for mapQ bias [0]*

*‐4 FLOAT min P‐value for end distance bias [0.0001]*

*‐e FLOAT min P‐value for HWE (plus F* *< 0) [0.0001]*

*‐p print filtered variants*

Step 6: Filtration of germline SNVs from tumour and cfDNA samples:

The VCF file of WBC (white blood cell) sample was used to filter germline SNVs from the VCF file of tumour and cfDNA samples using *‘bcftools isec’*

e.g., *bcftools isec GB13_Tumor.vcf GB13_WB.vcf‐p GB13_Tumor*

*bcftools isec GB13_cfDNA.vcf GB13_WB.vcf‐p GB13_cfDNA*

After this filtration, SNVs records that are only private to *GB13_Tumor.vcf or GB13_cfDNA.vcf* were used for the downstream analysis. SNVs records shared by *GB13_Tumor.vcf and GB13_WB.vcf or GB13_cfDNA.vcf and GB13_WB.vcf* were not used for downstream analysis.

Step 7: Calculating common SNVs between tumour and cfDNA samples:

After removing the germline SNVs from the tumour and cfDNA samples, we compared VCF files of tumour and cfDNA samples to extract common SNV records using *‘bcftools isec’*

e.g., *bcftools isec GB13_Tumor_Filtered_Germline.vcf GB13_cfDNA_Filtered_Germline.vcf‐p GB13_Tumor_cfDNA*

Further, germline variants identified in PBMC DNA were removed from the respective patient tumour and cfDNA variants and were considered as somatic variants.

### 2.10 Annotating final SNVs

Somatic variants from cfDNA and tDNA were annotated using the standalone Ensembl Variant Effect Predictor (VEP) pipeline (120). Annotation of the final VCF files with common SNVs in tumour and cfDNA samples was performed using the *‘VEP’* standalone pipeline:

e.g., *vep ‐i GB13_Tumor_Common.vcf.tsv ‐‐everything ‐‐cache ‐‐force_overwrite ‐‐filter_common ‐‐fork*

## 3 Results

### 3.1 cfDNA concentrations are elevated in the plasma of GBM patients

**Fig. 2:**
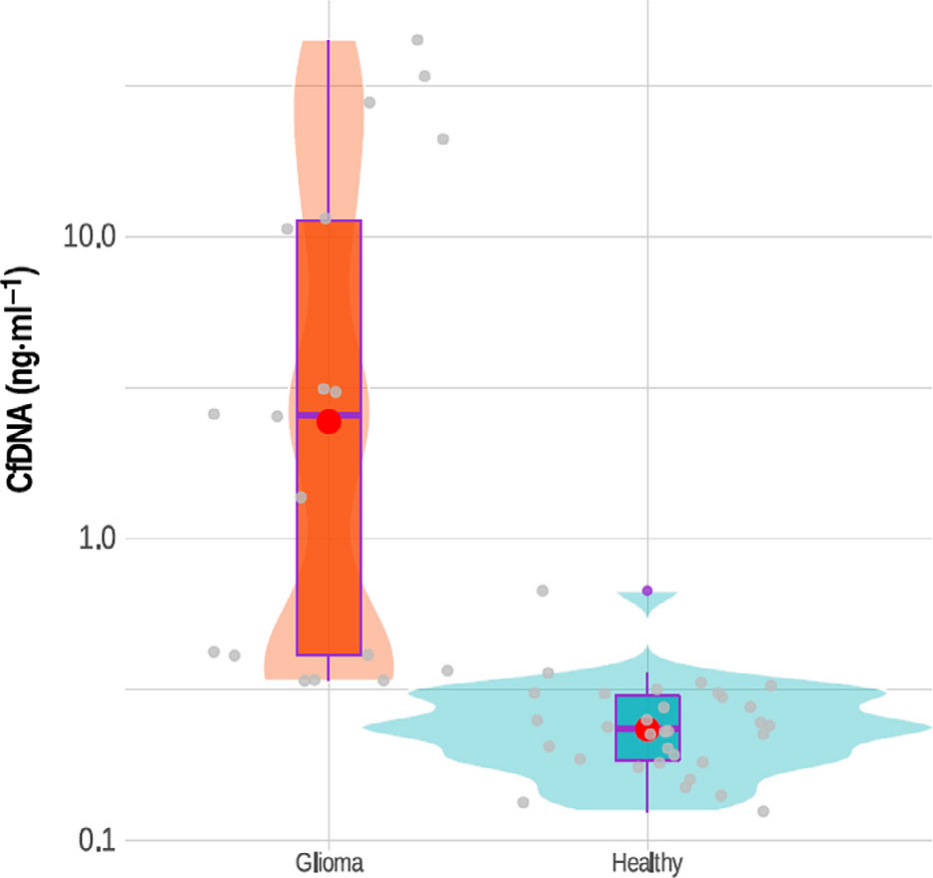
Quantification of cfDNA concentration in GBM patients *vs* healthy controls. The concentration of cfDNA isolated from 25 plasma samples of GBM patients was measured as described in the materials and methods. Violin plots represent 25 samples of patients *vs* 25 healthy controls cfDNA concentrations. The boxplots represent the confidence intervals for the samples *vs* controls; the red dots represent the median for both groups.

We hypothesized that cfDNA concentrations might differ between individuals with GBM and those assigned to a noncancer cohort. Thus, we obtained from tumour biobanks 25 blood samples that were collected from patients with GBM prior to surgery, and their corresponding samples of the resected tumours (Table 1 and Table S1). In addition, we collected 25 blood samples from healthy controls matching the ages of the GBM cohort. For each patient, cfDNA from plasma, genomic DNA (gDNA) from white blood cells (WBs), and tumour DNA (tDNA) from tumour tissues was extracted; fragments of sizes corresponding to cfDNA were identified and their concentrations were evaluated (Fig. 1). We assessed cfDNA plasma concentrations in the control cohort as ranging from 0.01 to 7.62 ng per mL of plasma. Next, we isolated detectable cfDNA from GBM samples and found that the cfDNA concentrations ranged between 12.6 and 137 ng per mL of plasma (Fig. 2). Thus, all GBM samples contained higher cfDNA concentrations than those of the control group (*P* < 0.0001, *t*‐test). We then examined the sizes of cfDNA molecules in all samples. A Bioanalyzer DNA High Sensitivity assay showed that in both GBM and healthy control samples, a cfDNA major peak was detectable at, or close to, 166 bp, which accounted for 85% of the circulating cfDNA. A smaller peak at, or close to, 332 bp accounted for 10% of the cfDNA and another peak at 2000–10 000 bp constituted 5% of cfDNA and likely represent fragments released by necrotic tissue (Fig. 1). Thus, liquid biopsy can generate high‐quality results, enabling analysis of cfDNA that was likely derived from apoptotic rather than necrotic cells. Our results indicate that the plasma cfDNA concentrations segregate GBM patients from healthy controls.

**Table 1:** Characteristics of GBM patients and tumour genomic alterations, as reported by the treating institution. MGMT‐ *O* ^6^‐methylguanine DNA methyltransferase; UM, unmethylated; M‐methylated; NA, not available; TERTp, telomerase reverse transcriptase promoter; WT, wildtype.

Biobank number	Age	Gender	IDH1/2	Other genomic alterations		Status
Hospital: Rabin Medical Center, Israel						
100058	62	Female	WT	NA		Dead
100067	71	Female	WT	TERTp mutation C228T	NA	Dead
100077	62	Male	IDH1m	TERTp mutation C228T	NA	Dead
100156	72	Female	WT	MGMT‐UM, TERTp WT, BRAF WT	7p and 7q gain, 10p and 10q loss, 9p loss, CDKN2A homozygous deletion, EGFR amplification	Dead
100101	51	Male	WT	MGMT‐UM, TERTp WT	NA	Dead
100106	55	Female	IDH1m	MGMT‐M, TERTp WT, BRAF WT	ATRX mutation, TP53 mutation, PTEN mutation	Alive
100142	76	Female	WT	MGMT‐M, TERTp mutation C250T, BRAF WT	TP53 mutation	Dead
100224	54	Male	WT	TERTp mutation C228T	NA	Dead
100237	75	Female	WT	MGMT UM, TERTp mutation C228T, BRAF WT	NA	Dead
100240	41	Male	WT	MGMT‐M, TERTp mutation C250T, BRAF WT	7p and 7q gain, 10p and 10q loss, EGFR amplification, TP53 mutation, PTEN mutation, CDK4 amplification	Dead
Hospital: Keil, Germany						
I	79	Male	WT	1p19q unknown, MGMT‐M		NA
II	54	Female	WT	MGMT‐M		Dead
IV	53	Male	WT	19q deleted, 1p intact, MGMT‐UM		NA
V	74	Male	WT	1p/19q not codeleted, MGMT‐M		NA
VIII	44	Male	WT	1p deleted, 19q intact, MGMT‐UM		NA
IX	57	Male	WT	1p/19q not codeleted, MGMT‐UM		NA
X	70	Female	WT	1p/19q not codeleted MGMT‐UM		NA
XI	80	Female	WT	MGMT‐M		Dead
XII	62	Male	WT	1p/19q not codeleted, MGMT‐UM		NA
Israeli National Tissue Bank (Midgham), Israel						
#1	77	Female	WT	MGMT‐UM, TERTp WT		Dead
#3	69	Female	WT	MGMT‐UM, TERTp WT		Dead
#5	53	Female	WT	MGMT‐UM, TERTp WT		Dead
#7	75	Female	WT	MGMT‐UM, TERTp WT		Dead
#13	71	Male	WT	MGMT‐UM, TERTp WT		Dead
#33	58	Male	WT	MGMT‐UM, TERTp WT		Dead

### 3.2 Mutation analysis of glioblastoma cfDNA data

**Fig. 3:**
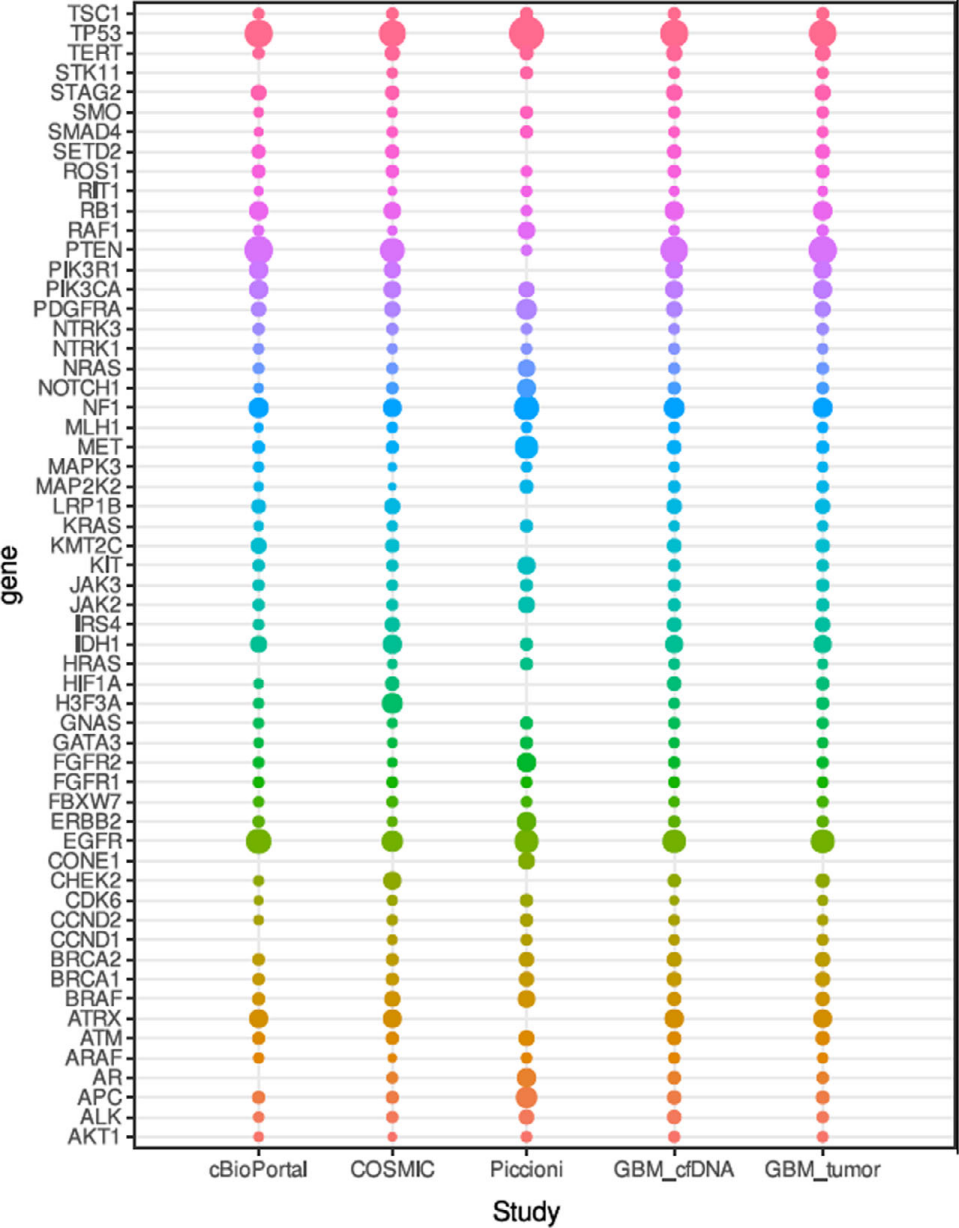
Variant calling analysis of cfDNA identifies high‐impact variants in patients with GBM. The circles diameter at the bubble plot describes the frequency for high‐impact mutations identified in GBM patients and in published cohorts. Gene names are represented at the y‐axes and the x‐axes describes the top‐50 mutated genes in GBM. All mutations statistics were collected for 50 cases in cBioPortal [45], all known mutations for GBM in the COSMIC [41], Piccioni et al. study [46], and cfDNA/tumour DNA from 25 samples in our study. Colors correspond to the ranks of the mutations from the higher ranked mutations on top to the lower ranked.

To confirm that the elevated cfDNA levels in the plasma of GBM patients was derived from tumour cells, we tested for the presence of mutations in both cfDNA and tDNA. We sequenced 25 cfDNA samples of GBM and 25 cfDNA samples from normal controls using a whole genome sequencing procedure (see Materials and methods) with 30× coverage (at least 150 million paired end [PE] 100 bp reads per sample). In addition, we sequenced tDNA (30× coverage, 150 million PE reads of 25 GBM tumour samples). We first removed all germline SNPs that appeared in patient gDNA using the variant calling method (see Materials and methods). Next, we sorted the mutations into “cfDNA only”, “tDNA only”, and “both cfDNA and gDNA” groups (Fig. 3). We found that GBM patients shared mutations in their cfDNA and tDNA, with 90% selectivity and 80% sensitivity (at 5% false discovery rate [FDR]). Variant calling analysis of gDNA was used to identify the background germline mutations of patients. We found a similar pattern of high‐impact alterations in both cfDNA and in tDNA in the 25 GBM patients (Table 2). These results indicate that in GBM, cfDNA includes molecular signatures that originate from the tumour mass.

**Table 2:** Average values of high‐impact alterations identified in cfDNA from 25 GBM patients.

Consequence type (Sequence Ontology term)	Average values of high‐impact alterations	
	Count	%
Splice donor variant	2	0.001
Splice acceptor variant	0.4	0.001
Stop‐gained	0.4	0.001
Missense variant	6.8	0.1
Splice region variant	14.0	0.15
Synonymous variant	7.0	0.001
5‐prime UTR variant	53.2	0.11
3‐prime UTR variant	318.8	0.82
Noncoding transcript exon variant	519.4	1.3
Intron variant	17981.6	47.5
Upstream gene variant	1662.6	4.28
Downstream gene variant	1429.6	4.0
TF binding site variant	101.0	0.3
Regulatory region variant	1823.4	4.5
Intergenic variant	13902.8	37.2

We extended our analysis to the top 50 genes that are most often mutated in GBM [41, 42, 43, 44, 45, 46]. For GBM patients, the distribution pattern of these mutations was highly conserved (Table 2). Of these 50 genes, 67% were identified as being mutated in the same precise genomic position in both cfDNA and tDNA, using at least five mapping reads sized 100 bp (Table 3). The mutated genes included *TP53,* which encodes a protein that is a tumour suppressor, and which is mutated in many cancers, including gliomas [46]. In addition to the most common GBM‐related genes, we also found mutations in the *BRAF* and *EGFR* genes, previously shown to be involved in glioma progression [46]. These results indicate that mutations found in cfDNA correspond to mutations in brain tumours with 95% specificity, allowing us to distinguish GBM at a 5% FDR, after removing the background noise of germline mutations (Table 3).

**Table 3:** Frequencies of high‐impact mutations identified in GBM patients and in published cohorts. Column #1 lists the top 50 genes found to be mutated in GBM. Columns #2, #3, and #4 present data on glioblastoma from three major studies [44, 45, 46]. The percentage indicates the frequency of the mutations.

Gene name	cBioPortal (585 patients)	Piccioni D.E.(419 patients)	TCGA/Pancancer (180 patients)	Our results for 25 GBM samples	
				Tumour	cfDNA
TP53	31.50%	58.70%	28.0%	30.0%	32.0%
IDH1	6.30%	2.00%	10.0%	8.0%	8.0%
PTEN	33.50%	0.80%	22.0%	33.0%	30.0%
EGFR	23.70%	20.00%	14.0%	20.0%	19.0%
H3F3A	0.80%	―	13.0%	2.0%	1.0%
PIK3CA	9.60%	5.00%	7.0%	9.0%	7.8%
ATRX	9.30%	—	9.0%	9.0%	10.0%
NF1	11.60%	22.90%	9.0%	11.0%	13.0%
BRAF	2.00%	7.00%	5.0%	3.0%	2.8%
RB1	9.60%	0.90%	7.0%	10.0%	9.7%
TERT	1.30%	2.80%	4.0%	4.5%	5.0%
PIK3R1	9.80%	—	6.0%	7.8%	7.0%
CHEK2	0.70%	—	8.0%	3.0%	2.1%
PDGFRA	4.00%	12.90%	5.0%	5.0%	5.1%
LRP1B	3.30%	—	5.0%	4.0%	4.3%
SETD2	2.80%	—	3.0%	3.5%	3.1%
STAG2	4.50%	—	3.0%	5.0%	5.2%
HIF1A	0.50%	—	3.0%	2.2%	3.1%
IRS4	1.00%	—	4.0%	4.0%	3.6%
KMT2C	4.80%	—	3.0%	3.0%	3.4%
MET	1.80%	19.00%	2.0%	2.0%	3.1%
APC	2.00%	14.00%	1.9%	2.5%	3.0%
AR	—	10.10%	1.1%	1.5%	2.3%
ERBB2	1.30%	10.10%	0.6%	1.3%	1.5%
FGFR2	1.00%	10.10%	0.5%	1.2%	1.3%
NOTCH1	0.50%	8.90%	1.5%	1.5%	2.0%
KIT	1.50%	8.00%	1.3%	1.5%	1.7%
NRAS	1.00%	7.00%	1.1%	1.5%	1.2%
RAF1	0.80%	7.00%	0.4%	1.2%	0.8%
CONE1	—	6.10%	—	—	—
JAK2	1.30%	6.10%	1.1%	2.1%	1.9%
ATM	1.80%	5.00%	2.1%	3.1%	2.8%
ALK	0.80%	4.00%	1.3%	1.3%	3.05%
BRCA1	1.50%	3.90%	2.0%	3.7%	3.5%
BRCA2	1.50%	3.90%	1.7%	3.8%	3.7%
MAP2K2	0.50%	2.80%	0.1%	1.5%	1.65%
CCND2	0.50%	2.00%	0.8%	0.8%	0.9%
CDK6	0.30%	2.00%	0.7%	0.7%	0.3%
GATA3	0.50%	1.90%	0.5%	0.9%	0.76%
GNAS	0.80%	1.90%	0.6%	1.2%	1.3%
HRAS	—	1.90%	0.5%	0.7%	1.1%
JAK3	1.30%	1.90%	1.0%	2.1%	1.9%
KRAS	0.50%	2.00%	0.8%	0.9%	0.8%
SMAD4	0.30%	1.90%	0.9%	1.0%	0.96%
SMO	0.50%	1.90%	0.7%	1.4%	1.43%
STK11	—	1.90%	0.8%	1.2%	1.3%
TSC1	1.00%	1.90%	1.5%	1.9%	1.8%
AKT1	0.50%	0.90%	0.2%	0.9%	1.2%
ARAF	0.80%	0.90%	0.2%	0.8%	1.2%
CCND1	—	1.10%	0.5%	1.1%	0.8%
FBXW7	0.80%	0.90%	1.1%	1.1%	0.8%
FGFR1	1.00%	1.10%	1.1%	1.0%	1.1%
MAPK3	0.80%	0.90%	0.2%	0.8%	0.78%
MLH1	0.30%	0.90%	0.9%	0.9%	1.2%
NTRK1	0.80%	0.90%	0.6%	0.8%	1.1%
NTRK3	1.30%	0.90%	1.3%	1.3%	0.9%
RIT1	0.30%	0.90%	0.2%	0.53%	0.5%
ROS1	2.50%	0.90%	2.2%	2.5%	2.1%

**Fig. 4:**
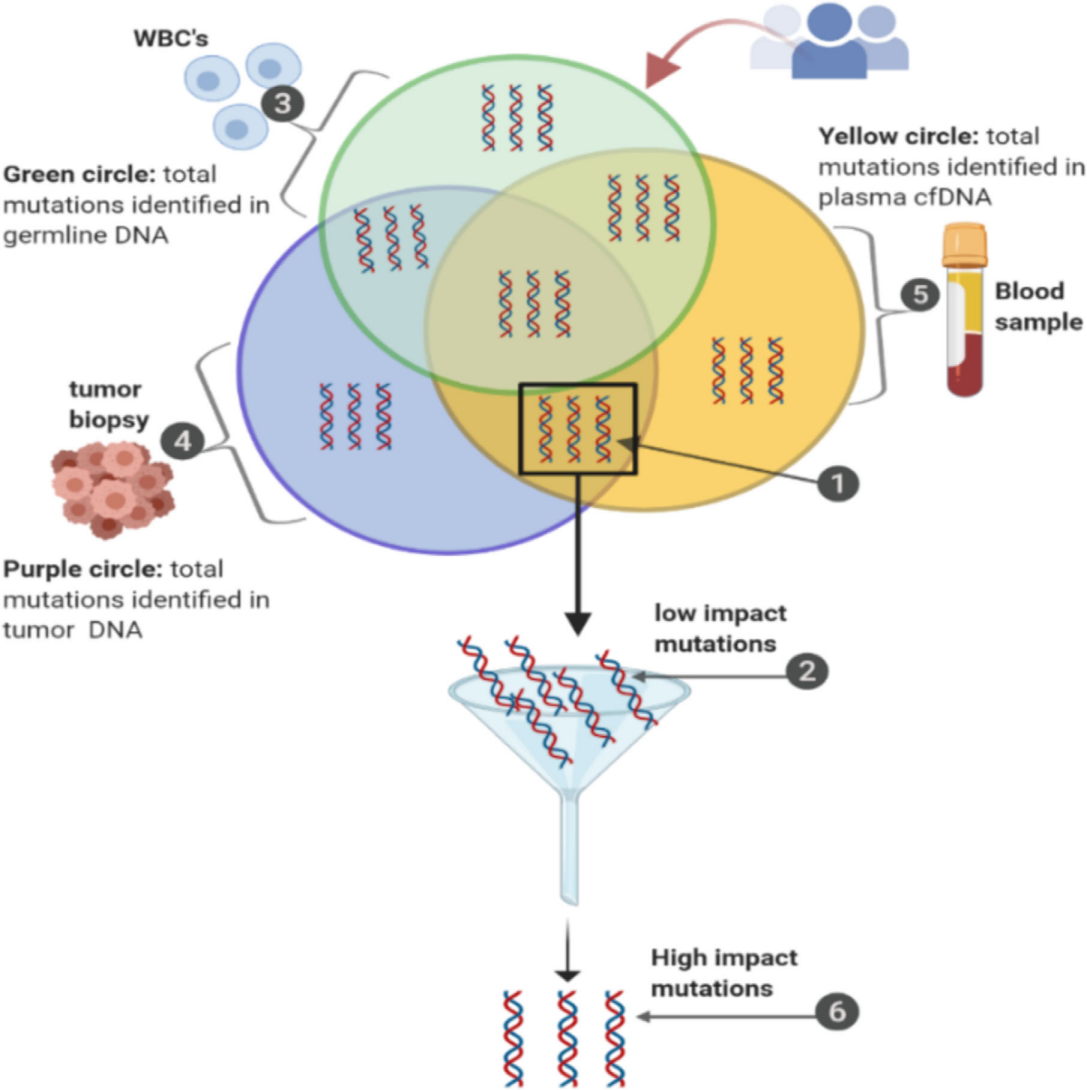
Schematic representation of the variant analysis method used to identify high‐impact variants. 1. Only somatic variants that were absent in germline DNA but commonly present in cfDNA and tDNA were selected. 2. From somatic variants, the low‐impact mutations were filtered. 3. The green circle represents the total number of variants detected in germline DNA of patients with GBM. 4. The blue circle represents the total number of variants detected in tumour DNA of GBM patients. 5. The yellow circle represents the total number of variants detected in the plasma cfDNA of GBM patients. 6. High‐impact variants were found.

**Fig. 5:**
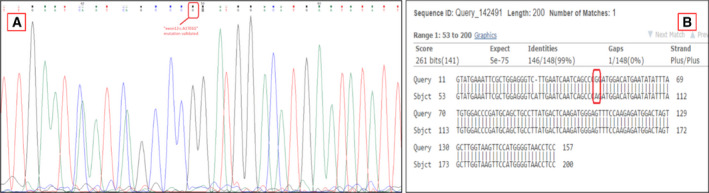
Mutation validation by Sanger sequencing. (A) Panel shows the Sanger sequencing raw results for a specific exon in the BCR/ABL chimera identified in the study. (B) Query sequence represents the known chimera sequence, and the subject represents the chimera identified for BCR/ABL in cfDNA of patient #100058 (Table 1).

As mentioned above, we compared somatic high‐impact mutations shared by cfDNA and tDNA in our patients with the mutation landscape data obtained from four studies [44, 45, 46] (Table 3 and Fig. 4). We validated these mutations by Sanger sequencing (Fig. 5), and found that cfDNA offered high‐level profiling of somatic mutations in all GBM patients. Specifically, we found mutations in genes that are strongly involved in GBM, i.e., *EGFR* (3’ UTR, intron, and downstream gene variants), *PDGFRA* (3’ UTR, intron and downstream and upstream gene variants), *PIK3CA* (intron and upstream gene variants), *PIK3R1* (upstream and downstream gene variants), and *TP53* (upstream gene, intron and downstream gene variants). Finally, we found that tumour‐suppressors were mostly absent in GBM due to missense mutations and that oncogenes appeared in the annotated data of mapped cfDNA sequences (data not shown). These results indicate that our liquid biopsy technique captures a broad spectrum of known glioma mutations at similar incidence rates as do standard tumour biopsies.

### 3.3 Fusion gene analysis and druggable fusions

We hypothesized that fusion genes contribute to glioma tumour formation, in addition to the point mutations described above, and that specific fusions, as opposed to mutation combinations, may be unique to different gliomas. To test this idea, we analysed cfDNA sequences from 25 control and 25 GBM samples, and from 180 TCGA GBM patients (downloaded from publicly available sources). We searched for fusions using our ChiTaRS 5.0 reference database (http://chitars.md.biu.ac.il/). We thus identified unique gene fusions, such as KDR‐PDGFRA (8%), and NCDN‐PDGFRA (40% of all samples) that correspond to the previously reported variations in PDGFRA in GBM. Interestingly, the PDGFRA protein fusions can be targeted by tyrosine kinase inhibitors, such as imatinib, sunitinib, and sorafenib [47, 48]. Moreover, we identified BCR‐ABL1 (8%), COL1A1‐PDGFB (8%), NIN‐PDGFRB (8%), and FGFR1‐BCR (4%), which can be targeted by imatinib, sunitinib, and sorafenib (Table 4, and Figs. [Link], [Link]). Also, ROS1 fusions were identified in 8% of patient cfDNA that might be targeted by analogues of crizotinib. These unique fusions were found in cfDNA and tDNA but not in the respective gDNA of the GBM patients and healthy controls with high read coverage (at least 10 reads mapping the junction site, 5% FDR) (Tables 4 and 5). These results indicate that a fusion gene signature may be readily detectable in GBM patients, thereby distinguishing them from noncancer controls.

**Table 4:** Druggable fusions observed in cfDNA of the 25 GBM patients in this study. The different colours indicate the sources of the samples as listed under the same sample ID in Table 1.

Biobank number	Observed fusions	Potential drugs
100058	BCR‐ABL	Imatinib, sunitinib and sorafenib
100067	KDR‐PDGFRA	Imatinib, sunitinib and sorafenib
100077	NCDN‐PDGFRA	Imatinib, sunitinib and sorafenib
100156	COL1A1‐PDGFB	Imatinib, sunitinib and sorafenib
100101	BCR‐ABL, KDR‐PDGFRA	Imatinib, sunitinib and sorafenib
100106	NA	NA
100142	NCDN‐PDGFRA	Imatinib, sunitinib and sorafenib
100224	FGFR1‐BCR	Imatinib, sunitinib and sorafenib
100237	CEP85L‐ROS1	Crizotinib, entrectinib and larotrectinib
100240	NCDN‐PDGFRA	Imatinib, sunitinib and sorafenib
I	NCDN‐PDGFRA	Imatinib, sunitinib and sorafenib
II	GOPC‐ROS1	Crizotinib, entrectinib and larotrectinib
IV	NIN‐PDGFRB	Imatinib, sunitinib and sorafenib
V	NA	NA
VIII	KDR‐PDGFRA	Imatinib, sunitinib and sorafenib
IX	NCDN‐PDGFRA	Imatinib, sunitinib and sorafenib
X	NA	NA
XI	COL1A1‐PDGFB, NCDN‐PDGFRA	Imatinib, sunitinib and sorafenib
XII	NIN‐PDGFRB	Imatinib, sunitinib and sorafenib
#1	GOPC‐ROS1	Crizotinib, entrectinib and larotrectinib
#3	NCDN‐PDGFRA	Imatinib, sunitinib and sorafenib
#5	NCDN‐PDGFRA	Imatinib, sunitinib and sorafenib
#7	CEP85L‐ROS1	Crizotinib, entrectinib and larotrectinib
#13	NCDN‐PDGFRA	Imatinib, sunitinib and sorafenib
#33	NCDN‐PDGFRA	Imatinib, sunitinib and sorafenib

**Table 5:** Druggable fusion genes and their targeting drugs identified in GBM samples archived in The Cancer Genome Atlas (TCGA) database.

Druggable Fusion genes	Targeting drugs	Junction type	Identified in glioblastoma patients or healthy controls
KMT2A‐FLNA	Daunorubicin	Intron‐exon	TCGA‐32‐1970 (tumour and germline DNA); TCGA‐06‐0157 (tDNA); TCGA‐27‐1831 (germline DNA); TCGA‐26‐5132 (tDNA); TCGA‐27‐2523 (tDNA); TCGA‐02‐2485 (germline DNA); TCGA‐26‐5135 (tDNA); TCGA‐06‐5411 (tDNA); TCGA‐15‐1444 (germline DNA)
FGFR1‐BCR	Dasatinib; Nilotinib; Ponatinib; Ruxolitinib; Imatinib; TKIs; Bosutinib; Sorafenib; AZD0530; AZD4547; BGJ398; Debio1347; Erdafitinib	Exon‐exon	TCGA‐06‐5411 (tDNA)
TPM3‐ROS1	Crizotinib, entrectinib, larotrectinib	Exon‐exon	TCGA‐15‐1444 (germline DNA)
TFG‐ALK	Crizotinib; entrectinib, larotrectinib Ceritinib; PF2341066; TAE684; novel ALK inhibitors; Alectinib; Brigatinib; Lorlatinib; foretinib	Exon‐exon	TCGA‐26‐5135 (tDNA)
MSN‐ALK	Crizotinib; entrectinib, larotrectinib, Ceritinib; PF2341066; TAE684; novel ALK inhibitors; Alectinib; Brigatinib; Lorlatinib	Exon‐exon	TCGA‐26‐5135 (tDNA)
MLLT1‐KMT2A	Daunorubicin	Exon‐exon	TCGA‐06‐5411 (tDNA)
BCR‐ABL1	Imatinib; Bosutinib; Dasatinib; Nilotinib; Ponatinib; Asciminib; TKIs; Sorafenib	Exon‐exon	TCGA‐27‐2523 (tDNA)
		Intron‐exon	TCGA‐15‐1444 (germline DNA)
NIN‐PDGFRB	Imatinib	Exon‐exon	TCGA‐02‐2485 (germline DNA);
AKAP9‐BRAF	Sorafenib; MEK inhibitors; Binimetinib + Encorafenib; Cobimetinib; Cobimetinib + Vemurafenib; Dabrafenib; Dabrafenib + Trametinib; Trametinib; Vemurafenib	Exon‐exon	TCGA‐06‐5411 (tDNA)
KMT2A‐MAML2	Daunorubicin	Exon‐exon	TCGA‐27‐1831 (germline DNA)
FGFR1‐PLAG1	AZD4547; BGJ398; Debio1347; Erdafitinib; Ponatinib	Exon‐exon	TCGA‐26‐5135 (tDNA)
KIF5B‐RET	Cabozantinib; Vandetanib	Exon‐exon	TCGA‐27‐2523 (tDNA), TCGA‐32‐1970 (tDNA)
EWSR1‐ATF1	PARP inhibitors	Exon‐exon	TCGA‐15‐1444 (germline DNA)
TPM3‐NTRK1	pan‐TRK inhibitor; Entrectinib; Larotrectinib; Crizotinib	Exon‐exon	TCGA‐26‐5132 (tDNA)
RARA‐PML	ATRA + arsenic trioxide	Exon‐exon	TCGA‐26‐5135 (tDNA)
GOLGA5‐RET	Cabozantinib; Vandetanib	Exon‐exon	TCGA‐27‐2523 (tDNA), GBM_#IA (cfDNA)
COL1A1‐PDGFB	Imatinib	Exon‐exon	TCGA‐26‐5135 (tDNA)
		Exon‐intron	TCGA‐32‐1970 (tDNA)
ABL1‐BCR	Imatinib; Dasatinib; Nilotinib; Ponatinib; Bosutinib; Ruxolitinib	Intron‐exon	TCGA‐15‐1444 (germline DNA)
FLI1‐EWSR1	PARP inhibitors; TK216	Intron‐exon	TCGA‐02‐2485 (germline DNA)
NPM1‐ALK	Crizotinib, entrectinib, larotrectinib	Intron‐exon	TCGA‐15‐1444 (germline DNA), Healthy‐Ctrl_#TS_0(cfDNA)
NIN‐PDGFRB	Imatinib	Exon‐exon	GBM_#GB7 (germline DNA), TCGA‐02‐2485 (germline DNA)
		Exon‐intron	TCGA‐26‐5132 (tDNA)
TENM4‐NRG1	Lapatinib	Intron‐exon	GBM_#GB3 (germline DNA)
SDC4‐ROS1	Crizotinib, entrectinib, larotrectinib	Exon‐exon	GBM_#VIIIA (cfDNA)

To study druggable targets, we analysed our next‐generation sequencing (NGS) datasets to identify hits among the 1207 predicted druggable fusions collected in the ChiTaRS 5.0 database [26]. Predicted druggable fusions are characterized by a preserved tyrosine kinase domain that can be targeted by specifically designed biologic drugs. We identified druggable fusions, particularly CEP85L‐ROS1 and GOPC‐ROS1, that bound crizotinib analogues (e.g., entrectinib and larotrectinib) in TCGA GBM patients, as reported previously by Davare et al. [49]. Interestingly, ROS1 fusions were mutually exclusive for EGFR and PDGFRA alterations in our patients, as previously reported [50]. Thus, we validated fusion *BCR‐ABL1* by PCR in two tDNA and corresponding cfDNA samples, as confirmed by cloning and Sanger sequencing. Finally, we validated *KDR‐PDGFRA* in three tRNA and cfDNA samples by PCR, cloning, and Sanger sequencing. Taken together, our results indicate that cfDNA may signal the presence of druggable gene–gene fusions that incorporate tyrosine kinases, and which can be possibly targeted by specific drugs. This will improve patient stratification in early‐phase clinical trials addressing potential novel GBM treatments.

### 3.4 Gene enrichment analysis

**Fig. 6:**
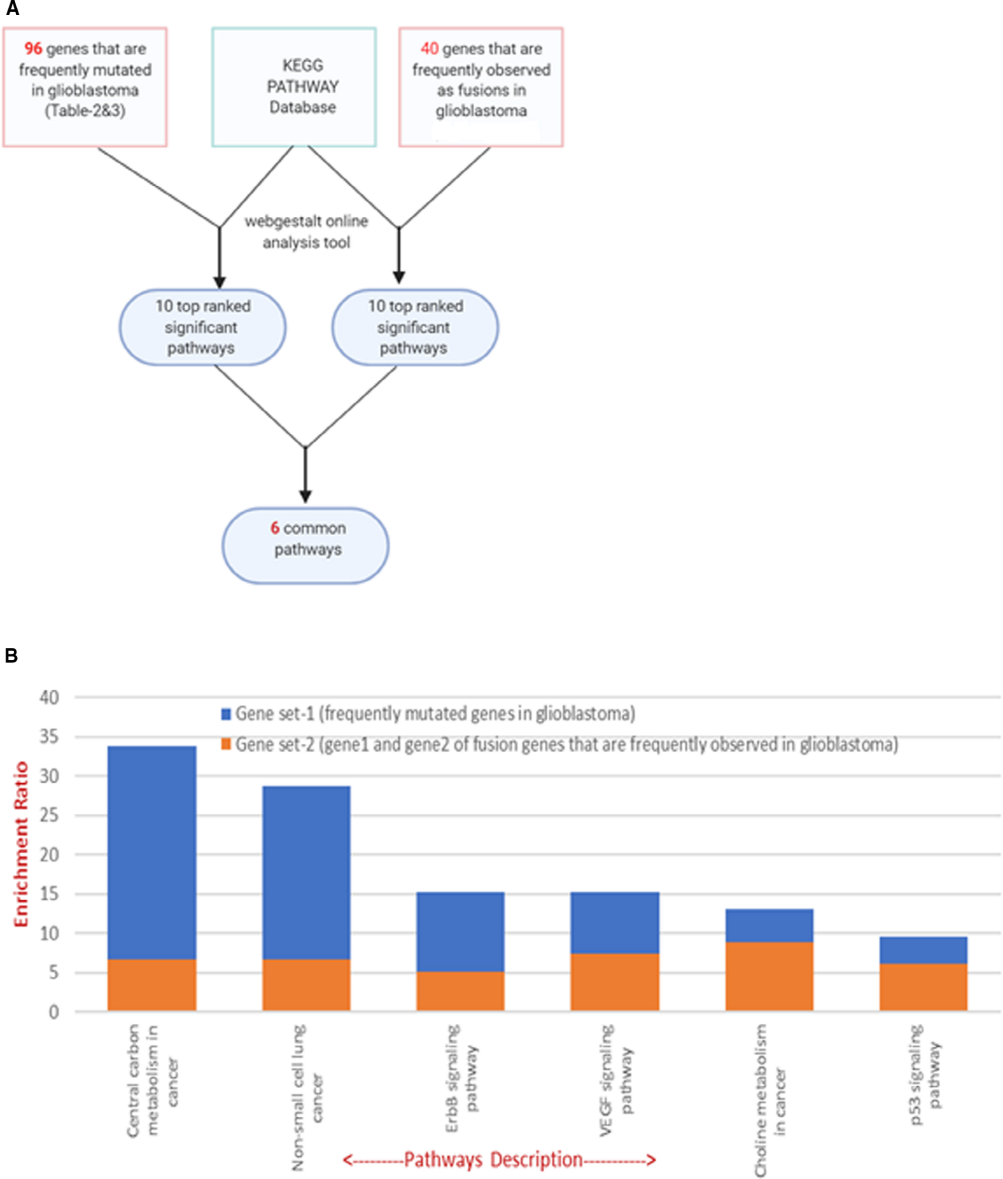
Gene set enrichment analysis. (A) Gene set enrichment analysis flowchart. A total of 96 genes identified as being frequently mutated in GBM patients, and 40 genes frequently observed as fusions in GBM patients were analysed against the KEGG human pathway database, using the online GSEA tool. (B) The bar graph shows six significant pathways, for which at least one gene was detected as both a frequently mutated glioblastoma gene and a gene identified as a frequent fusion in GBM.

Since functional mutations and fusions disrupt key metabolic pathways in cancer cells, we considered whether glioma‐specific pathway disruptions could be treated with targeted drug combinations. To test this possibility, we first found that a specific subset of fusions presented above and identified in cfDNA and tDNA encode druggable targets that are likely to respond to the crizotinib analogues entrectinib and larotrectinib and/or imatinib analogues (Tables 4 and 5). We subsequently hypothesized that pathways in gliomas were affected by mutations, as well as by fusions. We analysed the gene set and identified pathway enrichment for 96 genes that were previously reported as being frequently mutated in glioma patients [49, 50, 51] The KEGG PATHWAY [36, 37, 38] database was used for such analysis, with the most significant pathways being identified for each gene set (including the top 50 genes mutated in gliomas). The significant pathways for each gene set were then compared. Six significant pathways, namely, the ErbB signalling pathway, the VEGF signalling pathway, the choline metabolism pathway, central carbon metabolism in cancer, the p53 signalling pathway, and pathways in non‐small‐cell lung cancer were identified as common to the two gene sets (Fig. 6). Such analysis showed that cancer‐specific pathways are similar and targeted by either acquiring gene mutations or by forming gene–gene fusions. Thus, a comprehensive study of both gene mutations and gene–gene fusions can contribute to our understanding of targeted pathways in GBM patients.

## 4 Discussion

In this study we showed that plasma cfDNA concentration in GBM patients is higher than in healthy individuals. The direct association of cfDNA concentration and tumour stage was previously reported [8, 52, 53]. Moreover, the cfDNA concentration was shown to be a prognostic biomarker in colorectal, ovarian and breast cancers, non‐small‐cell lung cancer, and melanoma [8, 9, 10, 11, 52, 54, 55, 56, 57, 58]. Therefore, the cfDNA concentration can serve as a potential adjunct biomarker in GBM liquid biopsy for the diagnosis, prognosis, and possibly for prediction of high‐grade gliomas [46]. We extended these findings by addressing novel fusions and, particularly, druggable fusion targets, as shown in Tables 4 and 5.

In our 25 GBM patient samples, we detected the top 50 GBM mutations in cfDNA and showed that these are also found in tDNA. This suggests that liquid biopsy can provide information on the molecular signatures of GBM. It may serve as a noninvasive longitudinal diagnostic method for detection of molecular evolutions that occur during the disease. We also showed by gene enrichment analysis that these frequently observed fusion genes and the 50 most frequent genes from the glioma mutation landscape share common pathways that are substantial in GBM. These include the ErbB signalling pathway, the VEGF signalling pathway, the choline metabolism pathway, the central carbon metabolism pathway, and the p53 signalling pathway. The ErbB signalling pathway is enriched for both mutations and fusions in GBM. Receptor proteins ErbB1, ErbB2, ErbB3, and ErbB4 belong to the ErbB receptor family of tyrosine kinases. Upon ligand induction, these receptors activate downstream signalling pathways that lead to cell migration, cell proliferation, and antiapoptosis processes [59]. Mutations in these receptors lead to their constitutive activation, independent of ligand binding. Gene set enrichment analysis can compare two gene sets in GBM, namely, frequently mutated genes and frequently identified gene fusions. We found that gene fusions, together with mutations, directly target disease‐related pathways in GBM tumours.

Using our fusion gene database [17, 26, 60], we identified gene–gene fusions in cfDNA and tumour DNA, such as KDR‐PDGFRA (8%), and NCDN‐PDGFRA (40%) that correspond to the previously reported alterations of PDGFRA in GBM (43% of all our samples). The PDGFRA protein fusions can be targeted by tyrosine kinase inhibitors, such as imatinib, sunitinib, and sorafenib. Moreover, we identified BCR‐ABL1 (8%), COL1A1‐PDGFB (8%), NIN‐PDGFRB (8%), and FGFR1‐BCR (4%), which can be targeted by imatinib analogues (see protein domains observed in those fusions in Figs. [Link], [Link]). ALK and ROS1 fusions were also identified in 8% of patient cfDNA that might be targeted by analogues of crizotinib. Therefore, cfDNA may serve as a diagnostic tool for selecting the appropriate drug for individual patients. Targeted drugs with improved brain penetration should be tested accordingly, based on the dynamics of gene–gene fusions detected in patient blood, plasma, or serum samples.

## 5 Conclusions

We showed that liquid biopsy can play an important role in the molecular diagnosis of GBM, and as a potential means for selecting an accurate personalized approach for treatment of this devastating disease. The major advantage of liquid biopsy is its less invasive nature and its ability to provide information on a broad range of mutations and fusions in patients with brain tumours, while avoiding the need to perform invasive procedures to obtain tumour tissue for analysis. As therapeutic druggable fusion gene targets can be identified using liquid biopsy, this easy‐to‐use and noninvasive diagnostic technique will contribute to precise treatment of GBM patients at any stage of the disease.

## Conflict of interest

The authors declare no conflict of interest.

### Peer Review

The peer review history for this article is available at https://publons.com/publon/10.1002/1878‐0261.13157.

### Author contributions

MFM and TS designed, analysed, and supervised the project. VP and DRS produced all the experiments. MFM, AG, and RD produced bioinformatics analyses. AG provided the result visualization. SP, MK, and MVK ran library preparation. NGS analysed the study., RG, CF, AAK, YL, SHN, and ALB provided the tumour and blood samples of patients. VP, MFM, and TS wrote the article. All authors revised the article.

## Supporting information


**Fig S1**. Technical details related to the functional protein domains of BCR‐ABL1, BCR‐FGFR1, MSK‐ALK, TFG‐ALK, NPM1‐ALK, GOLGA5‐RET, AKAP9‐BRAF fusions observed in patients with GBM.

**Fig S2**. Technical details related to the functional protein domains of KIF5B‐RET, SDC4‐ROS1, TPM3‐ROS1, NIN‐PDGFRB, TPM3‐NTRK1, COL1A1‐PDGFB, EWSR1‐ATF1, KMT2A‐FLNA, KMT2A‐MAML2 fusions observed in patients with GBM. All the protein domains preserved in the sequence of the fusions have been mapped specifically to the reference human genome (query sequence) to show their potential druggable features.

**Table S1**. Age and gender details of the healthy controls used in this study.

**Supplementary Material** Detection of gene mutations and gene–gene fusions in circulating cell‐free DNA of glioblastoma patients ‐ an avenue for a clinically relevant diagnostic analysis

## Data Availability

All the data will be provided upon request.
