# Supplementary figures and images for: Detection of gene mutations and gene–gene fusions in circulating cell‐free DNA of glioblastoma patients: an avenue for clinically relevant diagnostic analysis
Source: Mol Oncol. 2022 Feb 11;16(10):2098–114. doi: 10.1002/1878-0261.13157 (PMC9120899; doi:10.1002/1878-0261.13157)

BCR\_\_ABL1 (AAL99544.1)

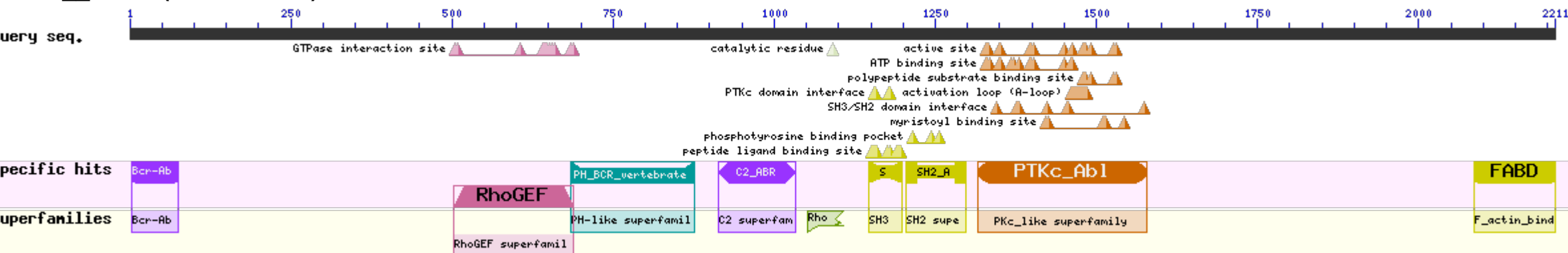

BCR\_\_ABL1 (AAA35697.1)

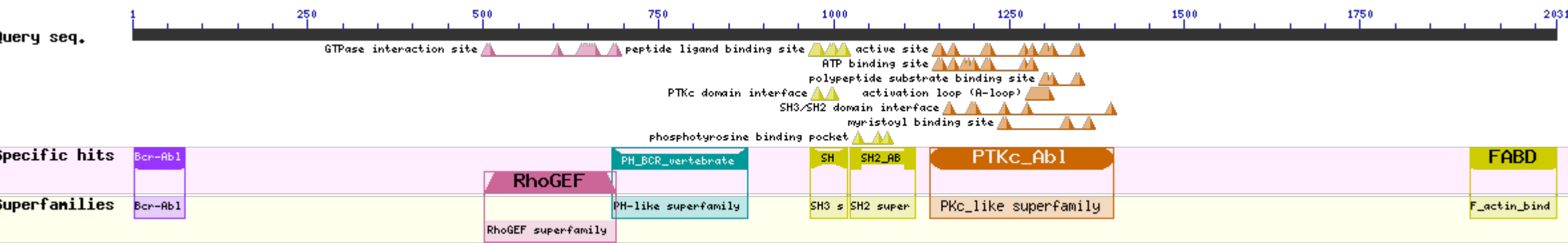

BCR\_\_FGFR1 (CAC82654.1)

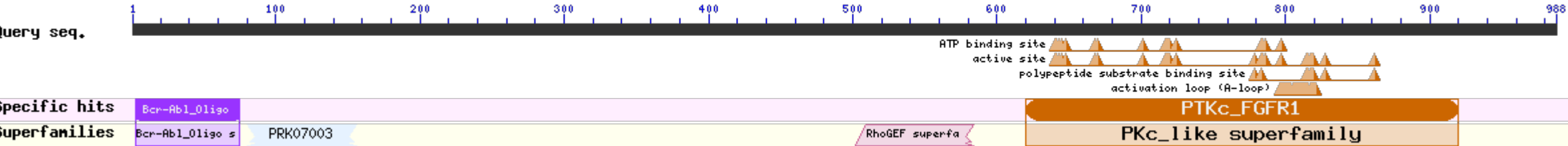

MSN\_\_ALK (AAK71522.1)

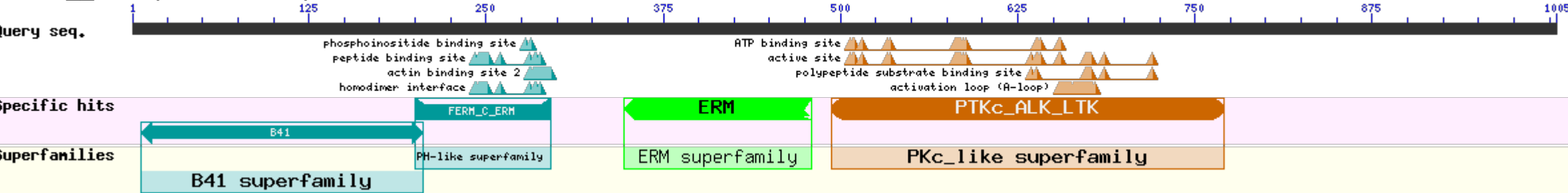

TFG\_\_ALK (AAF27292.1)

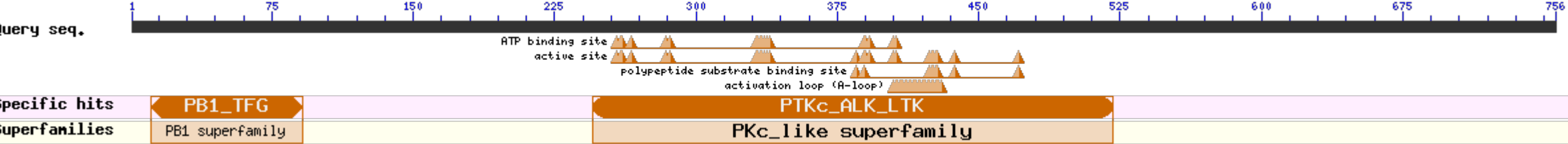

NPM1\_\_ALK (BAA08343.1)

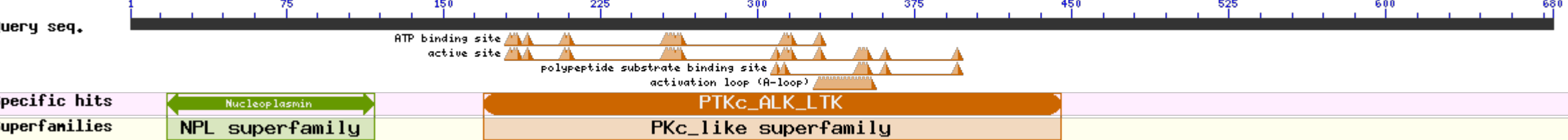

GOLGA5\_\_RET (CAA33787.1)

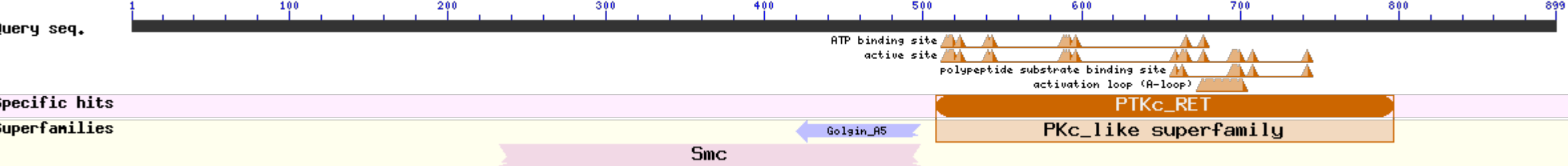

AKAP9\_\_BRAF (AAW30454.1)

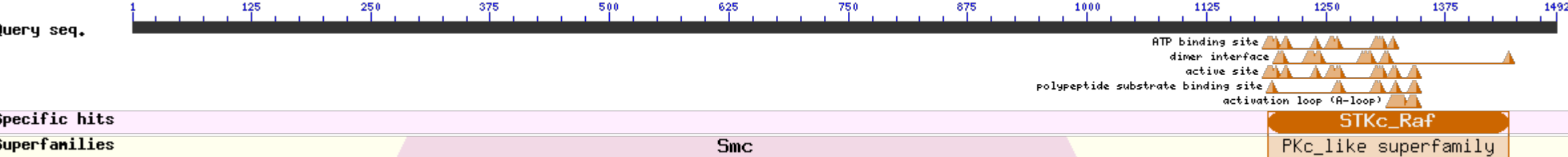

Supplement: Supplementary file 1 — Fig S1. Technical details related to the functional protein domains of BCR‐ABL1, BCR‐FGFR1, MSK‐ALK, TFG‐ALK, NPM1‐ALK, GOLGA5‐RET, AKAP9‐BRAF fusions observed in patients with GBM. [file MOL2-16-2098-s001.pdf]

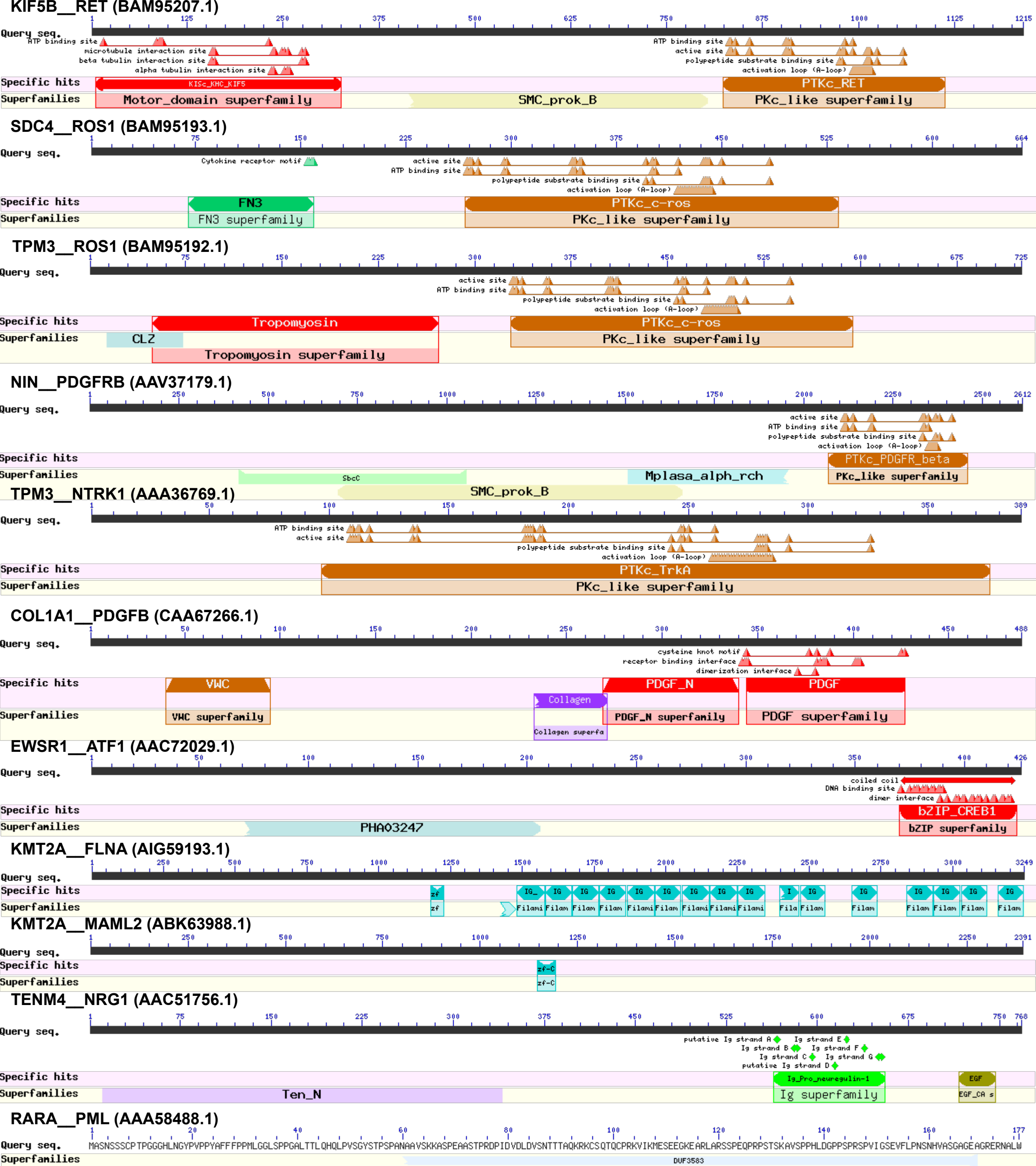

Supplement: Supplementary file 2 — Fig S2. Technical details related to the functional protein domains of KIF5B‐RET, SDC4‐ROS1, TPM3‐ROS1, NIN‐PDGFRB, TPM3‐NTRK1, COL1A1‐PDGFB, EWSR1‐ATF1, KMT2A‐FLNA, KMT2A‐MAML2 fusions observed in patients with GBM. All the protein domains preserved in the sequence of the fusions have been mapped specifically to the reference human genome (query sequence) to show their potential druggable features. [file MOL2-16-2098-s003.pdf]
