# Supplementary material for: Detection of gene mutations and gene–gene fusions in circulating cell‐free DNA of glioblastoma patients: an avenue for clinically relevant diagnostic analysis
Source: Mol Oncol. 2022 Feb 11;16(10):2098–114. doi: 10.1002/1878-0261.13157 (PMC9120899; doi:10.1002/1878-0261.13157)
Supplement: Supplementary file 4 — Supplementary Material Detection of gene mutations and gene–gene fusions in circulating cell‐free DNA of glioblastoma patients ‐ an avenue for a clinically relevant diagnostic analysis [file MOL2-16-2098-s004.pdf]

Supplementary Material

Detection of gene mutations and gene-gene fusions in circulating cell-free DNA of glioblastoma patients - an avenue for a clinically relevant diagnostic analysis

Vikrant Palande[1,#], Tali Siegal[2,#], Rajesh Detroja[1,#], Alessandro Gorohovski[1], Rainer Glass[3], Charlotte Flueh[4], Andrew A. Kanner[5], Yoseph Laviv[5], Sagi Har Nof[6], Adva Levy-Barda[6], Marcela Viviana Karpuj[1], Marina Kurtz[1], Shira Perez[1], Dorith Raviv Shay[1], Milana Frenkel-Morgenstern[1,7,*]

[1] Azrieli Faculty of Medicine, Bar-Ilan University, Safed 1311502, Israel

[2] Neuro-Oncology Center, Rabin Medical Center, 4941492, Petach Tikva, Israel and Hebrew University, Jerusalem, Israel

[3] Department of Neurosurgery, Ludwig-Maximilians-University, 81377 Munich, Germany

[4] Department of Neurosurgery, University Hospital of Schleswig-Holstein, Campus Kiel, 24105 Kiel, Germany

[5] Department of Neurosurgery, Rabin Medical Center, Petach Tikva 4941492, Israel and Sackler School of Medicine, Tel Aviv University, Tel Aviv, Israel

[6] Biobank, Department of Pathology, Rabin Medical Center, Petach Tikva 4941492, Israel

[7] The Dangoor Centre For Personalized Medicine, Bar-Ilan University, Ramat Gan, 5290002, Israel

[#]Vikrant Palande, Tali Siegal and Rajesh Detroja contributed equally to this study

*To whom correspondence should be addressed.

Tel: +972 (0)72-264-2901; Fax: +972 (0)72-264-2901; Email: milana.morgenstern@biu.ac.il

Gene/fusion-gene name	BCR-ABL1	
Forward- Green<br><br>Reverse- Red<br><br>Fusion junction- Yellow	>AB069693<br>CTCTGCTCTACAAGCCTGTGGACCGTGTGACGAGGAGCACGCTGGTCCTCCATGACTTGC<br>TGAAGCACACTCCTGCCAGCCACCCTGACCACCCCTTGCTGCAGGACGCCCTCCGCATCT<br>CACAGAACTTCCTGTCCAGCATCAATGAGGAGATCACACCCCGACGGCAGTCCATGACGG<br>TGAAGAAGGGAGAGGGAGAAGACAGGATGAAAGCTTCATCAACGAGGAAGAGATTACTCC<br>TTATGGAAGAAGCCCTTCAGCGGCCAGTAGCATCTGACTTTGAGCCTCAGGGTCTGAGTG<br>AAGCCGCTCGTTGGAACTCCAAGGAAAACCTTCTCGCTGGACCCAGTGAAAATGACCCCA<br>ACCTTTTCGTTGCACTGTATGATTTTGTGGCCAGTGGAGATAACACTCTAAGCATAACTA<br>AAG	
Forward primer	TGTCCAGCATCAATGAGGAG	T_m – 54.6

Reverse primer	GTTGGGGTCATTTTCACTGG	T_m – 54.1
Product size	230bp	

Sample ID	Sample
GBM patient #I	Tumor DNA
GBM patient #II	Tumor DNA
GBM patient #IV	Tumor DNA
GBM patient #IX	Tumor DNA
GBM patient #V	Tumor DNA
GBM patient #VIII	Tumor DNA
Positive control	CMV+GFP
Negative control	No template

Reaction mix

Total Reaction Volume	25 µl
2X ReddyMix PCR master mix	12.5 µl
Forward Primer (10 µM)	1.25 µl
Reverse Primer (10 µM)	1.25 µl
Template DNA	Varies
Nuclease free water	Varies

PCR conditions

Initial denaturation	95 ˚C	2 mins	
Denaturation	95 ˚C	25 sec	30 cycles
Annealing Gradient Tm-1	50.6 ˚C	35 sec	
Extension	72 ˚C	60 sec	
Final extension	72 ˚C	5 mins	
Hold	12 ˚C	∞	

Agarose gel electrophoresis (2%)

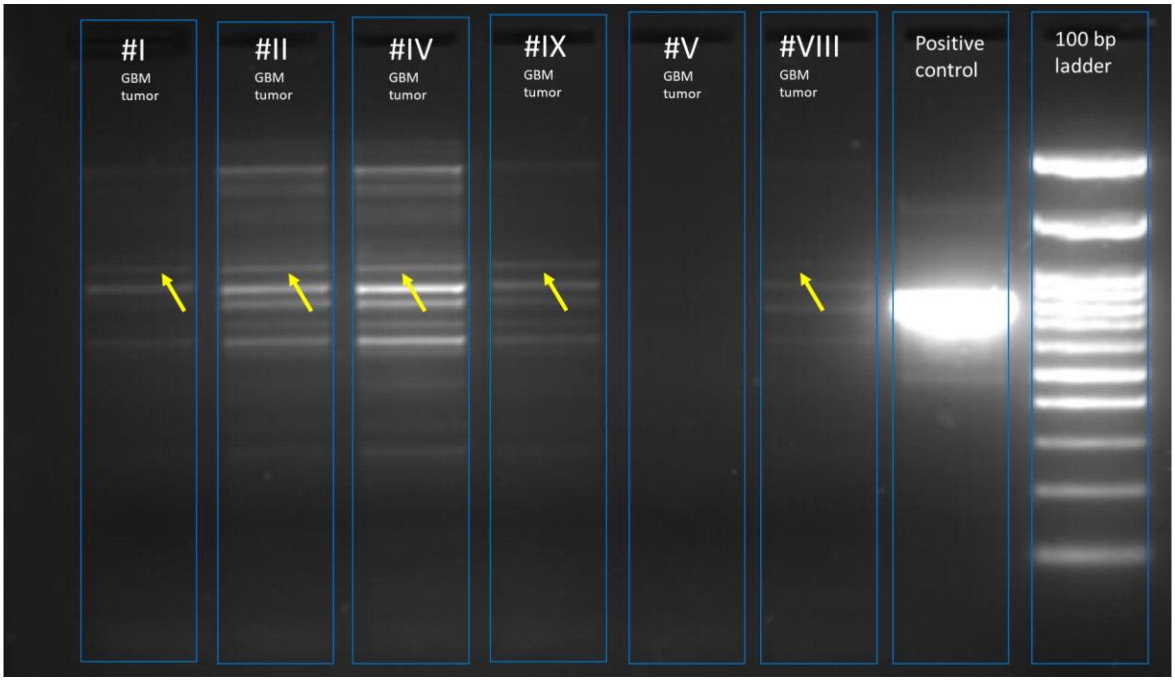
#I
GBM tumor
#II
GBM tumor
#IV
GBM tumor
#IX
GBM tumor
#V
GBM tumor
#VIII
GBM tumor
Positive control
100 bp ladder
